# Perceptions and experiences of healthcare professionals in implementing an ERAS protocol for minimally invasive heart valve surgery: a qualitative analysis

**DOI:** 10.3389/fcvm.2025.1407284

**Published:** 2025-02-21

**Authors:** Mona Elisabeth Schmid, Helene König, Jannik Stumm, Evaldas Girdauskas

**Affiliations:** Department of Cardiothoracic Surgery, University Hospital Augsburg, Augsburg, Germany

**Keywords:** enhanced recovery after surgery, minimally invasive cardiac surgery, heart valve surgery, ERAS implementation, experiences of healthcare professionals, qualitative analysis

## Abstract

**Introduction:**

ERAS protocols have revolutionized perioperative care by optimizing patient outcomes after major surgeries. Despite widespread recognition in various surgical specialties, the implementation of ERAS in cardiac surgery remains relatively novel.

**Methods:**

This qualitative study employed semi-structured interviews to explore the perspectives and experiences of healthcare professionals involved in ERAS implementation, focusing on identifying facilitators and barriers. The data collected from the interviews was subjected to qualitative content analysis to identify emerging themes and patterns.

**Results:**

The emerged themes highlight the importance of effective communication, interprofessional collaboration, and clear guidelines in facilitating ERAS implementation. While healthcare professionals universally recognized the benefits of ERAS for patient outcomes, they also identified challenges such as time constraints and resource allocation.

**Conclusion:**

The study emphasizes the need for effective communication, interprofessional collaboration, and clear guidelines to overcome the challenges of implementing ERAS. The importance of involving diverse healthcare professionals in ERAS development and implementation to enhance team spirit, motivation, and overall success is highlighted by our findings. These findings contribute to the ongoing refinement of ERAS protocols, ultimately improving patient outcomes in cardiac surgery and beyond. Future research should focus on longitudinal studies, collaboration across institutions, and exploration of implementation strategies.

## 1 Introduction

Enhanced Recovery After Surgery (ERAS) protocols have transformed perioperative care by providing a comprehensive and evidence-based approach to optimize patient outcomes after major surgeries. The primary objective is to reduce surgical stress, minimize complications, and expedite postoperative rehabilitation (1). ERAS protocols have gained widespread recognition across various surgical specialties (2). While ERAS protocols have been successfully implemented in other surgical areas, such as colorectal and orthopedic surgery, their application in cardiac surgery is still relatively new. The integration of ERAS principles into cardiac surgery, which is known for its complexity and postoperative challenges, presented both unique obstacles and opportunities. In the early 2000s, institutions and clinicians began adapting ERAS frameworks to meet the specific needs of cardiac surgery patients, which was a significant milestone in perioperative care (3). In 2017, the ERAS Cardiac Society was established to provide evidence-based guidelines for implementing ERAS programs in cardiac surgery, further solidifying these efforts (4).

ERAS emphasizes collaborative care across diverse healthcare disciplines to deliver holistic patient care. However, many existing guidelines focus solely on the involvement of physicians, disregarding the valuable contributions of other allied health professionals. Studies conducted by Martin et al. (5) and Seow-En et al. (6) shed light on the experiences of healthcare professionals involved in the care process and emphasized the necessity of broader interprofessional involvement in ERAS implementation. It is worth noting that the ERAS guidelines for neonatal intestinal surgery are the only ones that included nursing staff in their development (5, 6). All other ERAS guidelines were developed by physicians from different disciplines. The ERAS guidelines for cardiac surgery released in 2019 were conceptualized by physicians involved in the cardiac surgical care, including cardiac surgeons, anesthesiologists, and intensivists (4). However, nursing staff, physiotherapists, psychologists and other professional groups were not actively included in the guideline's writing committee. In contrast, Petersen et al. included physiotherapists in the development of their ERAS guidelines for minimally invasive heart valve surgery (7).

To address this implementation gap and fully embody the interprofessional ethos of ERAS, we aim to incorporate the opinions and expertise of all relevant healthcare professions included in our ERAS model. This multiprofessional approach was implemented at our university hospital in 2021. Our ERAS model includes following professions: cardiac surgery, anesthesia, physiotherapy, psychosomatics, ERAS nursing, general nursing, intermediate care unit (IMC) physicians and nursing, rehabilitation management, as well as other stakeholders such as research associates and project managers. By embracing a multiprofessional approach, we seek to develop a comprehensive ERAS model tailored to the unique needs of patients undergoing minimally invasive heart valve surgery. It is crucial to acknowledge that ERAS protocols must also be appropriate for the healthcare professionals, who play a vital role alongside the patients in ensuring the success of the perioperative care pathway (8, 9). To successfully implement ERAS, it is essential for professionals involved to be convinced of the ERAS concept and to fully embrace the interprofessional approach. This entails a commitment to learning from one other, providing mutual support, and promoting collaboration to optimize patient care and outcomes. Furthermore, incorporating the experiences of healthcare professionals in the implementation of ERAS protocols can offer several benefits, such as enhancing protocol adaptation, improving protocol acceptance and adherence, identifying of barriers and facilitators, promoting of continuous quality improvement, building a culture of collaboration, and validating protocol efficacy.

By integrating diverse perspectives and expertise, we aim to advance the implementation of ERAS protocols in cardiac surgery. This will ultimately improve patient outcomes and foster innovation in surgical care delivery. A qualitative approach was applied, utilizing Kuckartz's content analysis (10), to gain a comprehensive understanding of healthcare professionals' perspectives and experiences, ultimately informing the refinement and enhancement of ERAS protocols in cardiac surgery. The research question is focused on identifying the key facilitators and barriers that healthcare professionals encounter when adhering to ERAS protocols in the context of cardiac surgery, and their perceptions of the implementation process. The studýs findings will inform the optimization of ERAS implementation strategies.

## 2 Methods

### 2.1 Study design

This qualitative study utilized a semi-structured interview approach to explore the perceptions and experiences of healthcare professionals who were involved in the implementation of ERAS protocols for minimally invasive heart valve surgery.

### 2.2 Participants

Participants from various healthcare disciplines involved in perioperative care at our university hospital were recruited. The study included healthcare professionals who were directly engaged in the implementation of ERAS protocols for minimally invasive heart valve surgery. The participants comprised cardiac surgeons, anesthesiologists, physiotherapists, psychosomatic counselors, ERAS nurses, general nurses, intermediate care unit (IMC) physicians and nursing staff, rehabilitation management personnel, as well as other stakeholders such as research associates and project managers.

To ensure a representative sample, we aimed to include individuals from each relevant profession involved in the ERAS implementation process. Candidates were selected based on their direct experience with ERAS protocols and their roles in patient care during the perioperative period. Written informed consent was obtained from all healthcare professionals who agreed to participate in the study. The employees' respective authorities also agreed to their participation during working hours, ensuring that the study was authorized by the relevant healthcare institution.

### 2.3 Data collection

Semi-structured interviews were conducted with participants to capture their perspectives, experiences, and insights regarding the implementation of ERAS protocols. The interview questions covered various aspects of ERAS implementation, such as the perceived usefulness of the protocol, the quality of interprofessional collaboration, communication effectiveness, challenges encountered, expectations, and suggestions for improvement. The interviews were conducted in person and were audio-recorded with participant consent. Data collection continued until data saturation was reached, ensuring that no new themes or findings emerged. The goal was to include at least one representative from each relevant profession involved in ERAS implementation—ideally more. In instances where only a single professional from a specific field was involved in the ERAS process, that individual was interviewed to ensure comprehensive coverage across disciplines.

### 2.4 Data analysis

The data analysis followed a qualitative content analysis approach, as outlined by Kuckartz (10). The audio recordings of the interviews were transcribed verbatim and imported into qualitative analysis software (MAXQDA) for systematic coding and thematic analysis. An initial coding framework was developed based on the research questions and emergent themes from the data. This framework was iteratively refined throughout the analysis process to accommodate new insights. Two independent researchers (MS and HK) coded the transcripts, ensuring a comprehensive analysis. They initially coded a subset of transcripts to establish a coding scheme, which was then applied consistently across all transcripts. Discrepancies in coding were resolved through discussion and consensus, enhancing the reliability and validity of the analysis.

To ensure transparency and replicability, we conducted regular team meetings to discuss the coding framework and emerging themes. This collaborative reflection allowed for the exploration of diverse interpretations of the data while grounding the final thematic analysis in the participants' voices and experiences regarding the implementation of ERAS protocols.

### 2.5 Ethical considerations

This study was approved by the ethical committee of the Medical Faculty of the Ludwig-Maximilians-Universtität München on 07/13/2022 (Project Number: 22-0464). Participants gave written informed consent.

### 2.6 Trustworthiness

To ensure the trustworthiness of the study findings, the researchers employed several strategies. Firstly, they established credibility through prolonged engagement with participants, member checking, and triangulation of data sources. Secondly, they enhanced transferability by providing detailed descriptions of the study context, participants, and data collection procedures. Additionally, they employed purposive sampling to capture diverse perspectives. Thirdly, dependability was ensured by thoroughly documenting the research process, including detailed records of data collection, analysis, and interpretation. Fourthly, confirmability was addressed through reflexivity and systematic, transparent data analysis techniques such as coding frameworks. Finally, to promote authenticity, rapport and trust were established with participants, and a supportive and nonjudgmental environment was created for data collection. The findings were presented objectively, respecting the participants' voices and experiences. These strategies enhance the trustworthiness of the research.

## 3 Results

### 3.1 Sample description

The interviews were conducted between August 2022 and January 2023. A total of 15 participants were interviewed, including 11 females and 4 males from five different disciplines and professions: nursing, physiotherapy, psychosomatics, rehabilitation management, physicians, and surgery. The age range of the participants was 23–57 years, and their total work experience ranged from 1 to 28 years. The length of the interviews ranged from 10 to 37 min. Further details on the study participants can be found in Table 1.

**Table 1:** Study sample.

No.	Profession	Workplace	Gender	Age	Years of work experience in total	Level of education
1	Nurse	Surgical ward	Female	38	18	Medium
2	Nurse	Surgical ward	Female	24	6	Medium
3	Nurse	Surgical ward	Female	23	7	Medium
4	Advanced practice nurse (ERAS nurse)	Department of Cardiothoracic Surgery	Female	28	5	High
5	Physiotherapy	Surgical ward	Male	32	7	Medium
6	Psychosomatic counsellor	Department of Cardiothoracic Surgery	Female	29	5	High
7	Cardiac surgeon	Surgical ward	Male	38	11	High
8	Cardiac surgeon	Surgical ward	Female	24	1	High
9	Rehabilitation Manager	University Hospital	Female	37	15	High
10	Physician	IMC	Female	48	20	High
13	Physician	IMC	Female	45	16	High
11	Nurse	IMC	Male	35	17	Medium
12	Nurse	IMC	Female	41	23	Medium
14	Nurse	IMC	Female	25	7	High
15	Physician	Rehabilitation Clinic	Male	57	28	High

### 3.2 Collected data

**Figure 1:**
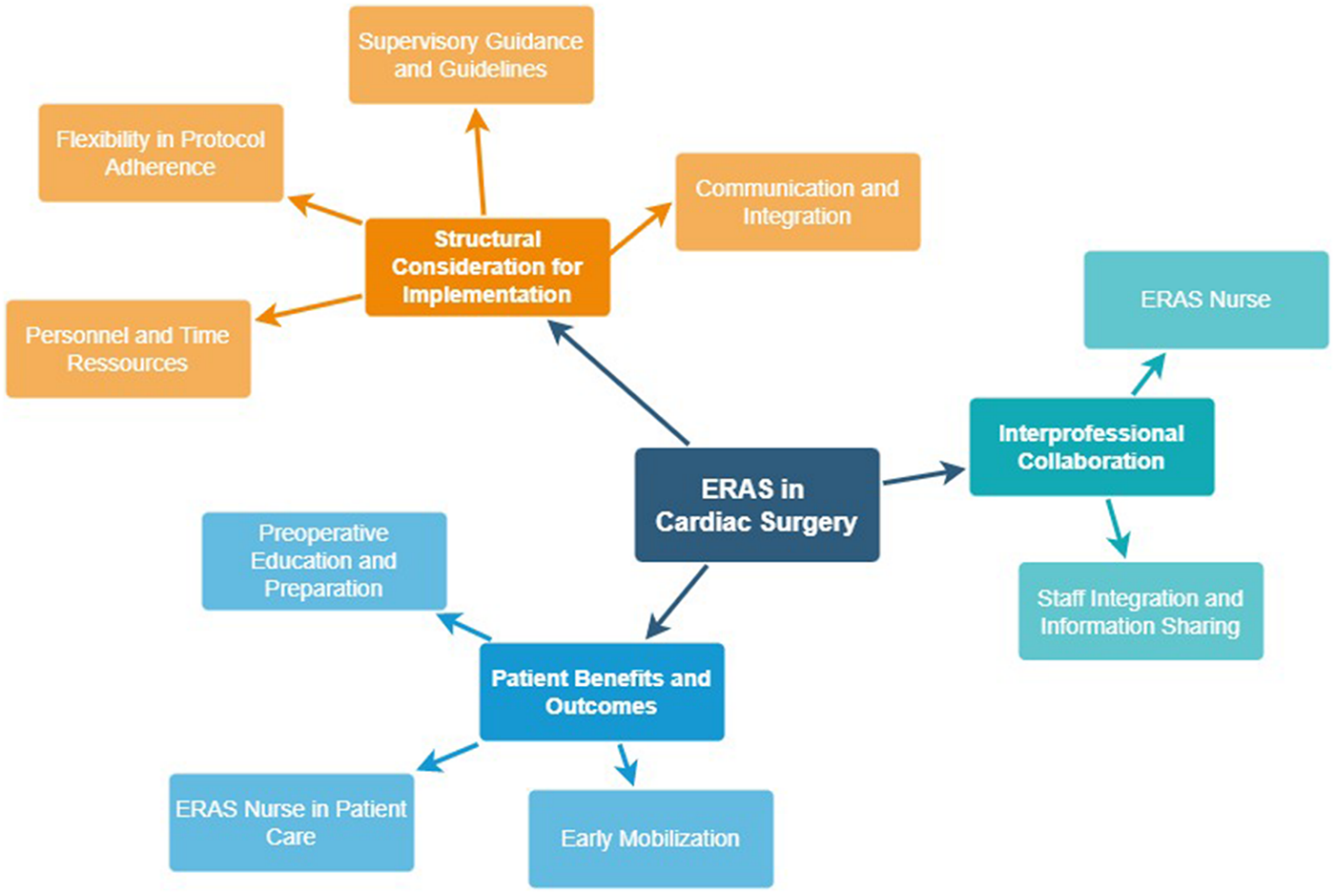
Mind map of categories.

The following three main categories were deductively extracted based on key elements of ERAS: (1) Structural considerations for implementation, (2) Interprofessional collaboration, and (3) Patient benefits and outcomes. Figure 1 displays the main themes with their corresponding subthemes.

Moreover Table 2 provides a more detailed summary of the subthemes, illustrating specific findings and representative quotes for each category.

**Table 2:** Summary of main themes, subthemes, and key findings.

Main theme	Subtheme	Key findings	Representative quote
Structural considerations	Personnel and time resources	Challenges due to personnel shortages and time constraints during ERAS implementation	“That time is not available to me as a regular nurse who still has twelve other patients.” (Nurse, Surgical Ward)
	Flexibility in protocol adherence	Importance of accommodating individual patient needs while adhering to ERAS protocols	“I do approach it very individually because some patients have no need, some just aren't interested, and some are fully engaged.” (Psychosomatics)
	Supervisory guidance and guidelines	Dedicated supervisors and clear guidelines were critical to implementation success	“We could just ask her anything. She informed us completely and simply explained the whole process.” (Nurse, Surgical Ward)
	Communication and integration	Effective communication among professionals is crucial for teamwork and motivation	“Communication with other healthcare professions, integrating them, and motivating them to participate is the biggest challenge.” (Cardiac Surgeon)
Interprofessional collaboration	ERAS nurse (Process coordinator)	ERAS nurse acts as a central figure, coordinating care and reducing staff workload	“Everything runs smoothly and efficiently through the ERAS nurse.” (Psychosomatics)
	Staff integration and information sharing	Interdisciplinary ward rounds promote collaboration but face scheduling challenges	“The problem is that in clinical practice, it's often difficult to make it happen.” (Cardiac Surgeon)
Patient benefits and outcomes	Preoperative education	Psychological preparation and organizational planning improve patient readiness	“The patient feels much better—that's what it's all about. Someone listens to them beforehand.” (Physician IMC)
	Role of ERAS nurse in patient care	Patients feel safer and supported with a dedicated ERAS nurse	“For them, the ERAS nurse was like an anchor. They received so much validation and structure from her.” (Cardiac Surgeon)
	Early mobilization	Early mobilization improves recovery, physical function, and reduces hospital stay duration	“Patients with ERAS are mobilized early, several times a day—very good for the patients.” (Physiotherapist)

The main statements of the participants presented in this chapter are supported by original quotes from the interviews. The interviews have been translated from German into English.

### 3.3 Structural considerations for implementation

#### 3.3.1 Personnel and time resources

The interviewed healthcare professionals highlighted the challenges related to personnel shortages and time constraints during the implementation of ERAS protocols. They identified limited staffing and time pressures as barriers to protocol adherence and effective patient care.

“Nevertheless, I believe that if one has a Fast-Track concept—which is very pleasant and beneficial for the patients, and therefore I am fully behind it—one will indeed have to make a greater effort, but there is also a greater benefit, yes.” (Physician IMC)

“The biggest issue for the [rehab management] in general is scheduling. That's crucial.” (Rehab Management)

Adhering to predefined structures and protocols is crucial for the ERAS program to function effectively. However, healthcare workers may face difficulties in doing so due to limited time resources.

“That time is not available to me as a regular nurse who still has twelve other patients (Nurse Surgical Ward)”

“There's a lot of pressure to do it because it's in the protocol [ERAS protocol].” (Nurse IMC)

#### 3.3.2 Flexibility in protocol adherence (patient centeredness)

Participants highlighted the value of flexibility in adhering to ERAS protocols to accommodate individual patient needs and preferences.

“But ultimately, I do feel that if you also take a bit of an individual approach to the patients and don't just rigidly follow a conversation guide, but rather look a bit at where the needs are for each patient, then I believe that everyone can take something positive home with them. […] And I do approach it very individually because some patients have no need, some just aren't interested, and some are fully engaged.” (Psychosomatics)

“And regarding the content, I would make it a bit more flexible. So that I can go more individually with the patient, that I don't have to check off this checklist for everything, but rather can see what does this person need.” (Psychosomatics)

“They [preliminary discussions] all worked well. Um, so it varies how much information they ultimately want. […] Make it shorter and look individually whether the patients have the need or not. There are many who also don't necessarily want these long conversations—and whether you couldn't maybe, partially do it in advance or online.” (Cardiac Surgeon)

A patient-centered approach was considered crucial for improving patient outcomes and satisfaction.

“And I think that's the main thing that the patients feel well taken care of.” (Physician IMC)

#### 3.3.3 Supervisory guidance and guidelines

The results showed that the support of dedicated supervisors and clear guidelines were critical to the successful implementation of ERAS. Supervisory guidance provided support and direction to the nursing team, ensuring consistency and adherence to protocol guidelines.

Interviewer:

“Did it also help that the [already experienced] ERAS nurse from Hamburg was there [before you started to implement ERAS]?”

Respondant:

“Yes, definitely, because we could just ask her anything. Because we couldn't really imagine much about ERAS. Like what should we pay attention to, what happens, how does it work? And she informed us completely and simply explained the whole process.” (Nurse Surgical Ward)

Additionally, the use of standard operating procedures (SOPs) and checklists was found to be beneficial in reducing the potential for errors.

“Everything is standardized. That's actually good; everyone knows what they need to do and in what sequence.” (Nurse IMC)

“So, for ERAS, there's this nice sheet outlining everything you do step by step. […] I think it's really well done; you can literally check off one point after another from top to bottom, cross things out, whatever you want, and then you don't forget anything.” (Nurse IMC)

#### 3.3.4 Communication and integration (motivation of professions)

Effective communication and integration among healthcare professionals are crucial factors for a successful ERAS implementation. Motivating and engaging all professionals involved in the care process promotes teamwork, collaboration, and a shared commitment to achieving ERAS goals.

“Exactly, communication is very important, and openness.” (Nurse Surgical Ward)

“The biggest challenge, I believe, is the communication with other healthcare professions, integrating them and motivating them to participate. Um, because they have few benefits from it […]. They usually have more effort and little benefit from it. That's the biggest factor—motivating other professions. And then having the logistics available—IMC, monitoring beds—[…] those are the biggest challenges. And of course, financing the staff.” (Cardiac Surgeon)

When communication is insufficient or missing, healthcare professionals become demotivated and do not feel sufficiently integrated into the implementation process. During the ERAS implementation, some participants failed to attend regular meetings for updates.

“I would like to know where we currently stand […] and have such a feedback discussion.” (Physician IMC)

### 3.4 Interprofessional collaboration

#### 3.4.1 ERAS nurse (process coordinator)

The ERAS nurse played a crucial role as a process coordinator in promoting interprofessional collaboration and coordinating patient care. As the main point of contact for almost all involved professions, they were always informed about the ERAS patients and possessed all relevant information.

“Everything runs smoothly and efficiently through the ERAS nurse” (Psychosomatics)

“It's actually more the ERAS nurse who is the contact person.” (Nurse Surgical Ward)

In addition, the ERAS nurse assisted other healthcare professionals, such as nurses and physiotherapists, in their duties, thereby helping to reduce or alleviate their workload.

“What I usually do alone [for the ERAS protocol]—it just takes ten minutes longer [then when it´s not an ERAS patient]. But that [the ERAS nurse] was really great support, I found. So for me, it was even more relaxed when I heard she was coming along.” (IMC Nurse)

“Yeah, she's a huge help. She takes care of the patients, the discharges. She also checks in on everyone regularly. For us, that's really a big relief”. (Nurse Surgical Ward)

#### 3.4.2 Staff integration and information sharing

Staff integration and information sharing enhanced interprofessional collaboration, facilitated by effective communication. Interprofessional ward rounds served as a positive example, promoting teamwork, coordination, and shared decision-making among healthcare professionals. However, planning and executing interprofessional ward rounds proved to be time-consuming and challenging.

 “I'm actually a big advocate of interdisciplinary rounds in general. I always think it's good when all the people involved in patient care are present. The problem is that in clinical practice, unfortunately, it's often difficult to make it happen.” (Cardiac Surgeon)

“The patient benefits from interprofessional ward rounds […]. When everyone sticks to meeting at a set time, then everyone can prepare for it and it works. The problem arises when certain parties don't stick to the schedule or to what has been agreed upon.” (Nurse Surgical Ward)

As previously mentioned, the ERAS nurse collaborates with other healthcare professionals, including psychosomatic specialists, to provide comprehensive patient care.

“I'm in very close communication with our ERAS nurse. […] I believe that we complement and support each other quite well. Sometimes the ERAS nurse may have a better rapport with the patient and takes on a few points from me, and sometimes it's the other way around. There's definitely a very close collaboration there.” (Psychosomatics)

All participants reported having access to the necessary patient information. However, some professionals expressed a desire for more involvement in feedback discussions and regular updates on ERAS protocols.

“I would like to know where we currently stand […] and have such a feedback discussion.” (IMC Physician)

### 3.5 Benefits and outcomes

#### 3.5.1 Preoperative education and preparation

Preoperative education and preparation, including psychosomatic support, were identified as essential components of ERAS protocols. Providing patients with comprehensive information and psychological support before surgery contributed to improved psychosomatic well-being and readiness for the surgical procedure.

“They are also excellently prepared psychologically for the surgery, […] they are usually very well adjusted to the situation they are in. […] There are generally many patients who could benefit greatly from elements of an ERAS model—especially this intensive physiotherapy, and particularly psychological support. I find that many patients after heart surgery are also […] psychologically very burdened.” (Cardiac Surgeon)

“The patient feels much better—that's what it's all about. Someone listens to them beforehand, they get a bit of homework—that makes the patient to be the process owner […] and from that perspective […]—I think that's great.” (Physician IMC)

ERAS patients receive an education session at least 2 weeks prior to surgery. This allows for earlier completion of organizational tasks, such as scheduling rehabilitation dates, resulting in a better-structured and planned process compared to standard care.

“For the patient […] it is definitely advantageous if they have a little longer to deal with it or even to deal with it at all. Are there other things they might need to organize at home as well?” (Rehab Management)

#### 3.5.2 ERAS nurse in patient care

The participation of an ERAS nurse in patient care was linked to favorable patient outcomes. The ERAS nurse served as a trusted individual for patients and acted as the primary point of contact.

“Because she was such a strong point of contact for the patients. […] for them, the ERAS nurse was like an anchor. They discussed every problem with the ERAS nurse, which was wonderful because they received so much validation and structure from her. And practically everything for these patients was caught, processed, cushioned, built up, and so on, down to the last detail, which is really positive.” (Cardiac Surgeon)

“I'm also a bit responsible for the patients, so to speak. I'm simply there to coordinate the whole process to make the whole thing, um, as good and pleasant as possible for patients. And to give them the information they need.” (ERAS nurse)

“Patients feel totally safe because they already know the people who they will see here again from the day before the surgery. And who will look after them afterwards. […] And I personally think that's very good, because building a relationship is one of the most important things when you're looking after a patient.” (ERAS nurse)

#### 3.5.3 Early mobilization

The implementation of early mobilization strategies as part of ERAS protocols proved effective in promoting postoperative recovery. Encouraging patients to mobilize early after surgery contributed to faster recovery, improved physical function, and shorter hospital stays.

“What really stands out to me is that while both groups undergo heart surgery, those who come directly after the surgery, even though being a bit weak from the surgery, can be mobilized just as quickly as those who had a conventional heart surgery. […] I find it fascinating that in terms of how quickly they can get up, they don't really differ. They're essentially identical, even though one group is freshly out of surgery and the other starts mobilization a few days later.” (Nurse IMC)

“They're discharged much faster because they're simply getting fit more quickly. But they also receive physiotherapy on weekends, which the others don't have.” (Nurse Normal Ward)

“Yes, as I said, the patients with the ERAS stuff are mobilized early, several times a day—I find that great, very good for the patients.” (Physiotherapist)

## 4 Discussion

The implementation of ERAS protocols in cardiac surgery represents a significant advancement in perioperative care. This study aimed to explore the perspectives and experiences of healthcare professionals involved in ERAS implementation, particularly focusing on identifying key facilitators and barriers.

The findings emphasize the importance of effective communication, interprofessional collaboration, and clear guidelines in facilitating the successful implementation of ERAS protocols. Healthcare professionals from various disciplines involved in perioperative care consistently underscored the benefits of ERAS for patient outcomes. However, our analysis identified several challenges and limitations that require careful consideration in future ERAS implementation endeavors.

In the following sections, we discuss the key themes that emerged from our analysis.

Effective communication emerges as a vital component in the implementation of ERAS. Research has consistently shown that miscommunication is associated with poor patient outcomes and delayed treatment (11). In line with this, several studies focused specifically on ERAS implementation (in cardiac surgery) further highlight the critical role of communication in achieving optimal outcomes and facilitating smooth processes (12–15). To enhance communication in ERAS implementation, several suggestions can be considered:

Implementing structured agendas and clear objectives for regular ERAS meetings can maximize efficiency and effectiveness. These meetings provide a dedicated platform for healthcare professionals to discuss ERAS-related matters and share updates. They also serve as valuable opportunities for continuous education and training on ERAS protocols, fostering a sense of community and teamwork among healthcare team members. Several studies have shown that communication among healthcare professionals can lead to misunderstandings due to differences in terminology, language, communication styles, and confidence levels (16–20). Therefore, regular training on effective communication techniques provides healthcare professionals with the skills needed to communicate efficiently within the interprofessional ERAS team. Moreover, Cornett & Kuziemsky recommend integrating protocols into meetings to support team communication. It is also crucial to establish common ground at the protocol, document, and terminology levels (19).

Standardized feedback processes should be developed to ensure consistency and follow-up on action items identified during feedback sessions (21). Moreover, the utilization of technology and digital platforms can facilitate communication and collaboration, particularly for remote or dispersed teams. However, organizing and maintaining these meetings may pose challenges, requiring additional time and resources, and attendance can be difficult due to competing priorities.

Interprofessional ward rounds facilitate interdisciplinary collaboration and shared decision-making in patient care. These rounds allow healthcare professionals involved in the patient's care pathway to communicate effectively, share information, and coordinate care plans (22, 23). However, interprofessional ward rounds can be time-consuming and disruptive to workflow if not well-organized or streamlined. Effective leadership and coordination are needed to ensure the participation and engagement of all relevant team members during these rounds (24).

In addition to interprofessional ward rounds, a key aspect of interprofessional collaboration is the role of the ERAS nurse as a process coordinator. The ERAS nurse plays a critical role in facilitating communication and coordination among healthcare professionals involved in the perioperative care of patients. By serving as a central point of contact, the ERAS nurse ensures that all members of the ERAS team are informed and aligned with the goals and protocols of the ERAS program. This coordination role extends to various aspects of patient care, including parts of preoperative education, perioperative management, and postoperative care. Through their expertise and leadership, ERAS nurses contribute significantly to the smooth implementation of ERAS protocols, the effective interprofessional collaboration and the optimization of patient outcomes (9, 25). Recent literature has reinforced the importance of the ERAS nurse in optimizing team collaboration and patient care (14, 15, 25, 26).

The presence of a dedicated supervisor (i.e., an experienced ERAS nurse) or ERAS centers of excellence in ERAS networks, staffed with experts from centers where ERAS is successfully implemented, can greatly facilitate ERAS implementation (24, 27). These entities provide guidance, support, and resources to healthcare teams, enabling the identification of clinic-specific challenges and the development of tailored solutions in partnership with experienced ERAS professionals. However, engaging ERAS experts and participating in ERAS networks can present challenges, including the time and resources required, logistical considerations, and funding issues.

In addition, providing standardized Standard Operating Procedures (SOPs), guidelines, and checklists is paramount to ensuring consistency and quality in the implementation of ERAS in different clinical settings (28). These resources serve as practical roadmaps that guide healthcare teams through the ERAS process, while allowing flexibility to accommodate the unique needs and resources of each setting. By maintaining standardized processes while adapting to local contexts, healthcare teams can optimize ERAS implementation and improve patient outcomes.

A significant challenge facing healthcare systems worldwide is the shortage of human resources (29). ERAS programs offer a promising solution to alleviate some of these challenges by optimizing resource utilization, improving efficiency through standardized processes, and enhancing team morale and productivity through staff training and development (30, 31). While ERAS programs may require additional work upfront, the long-term benefits often outweigh the initial investment. As healthcare professionals become more familiar with ERAS protocols and teamwork improves, the workload associated with ERAS implementation tends to decrease over time (32). This reduction in workload frees up resources and allows healthcare professionals to focus on other critical areas of patient care.

Moreover, investing in staff training and development as part of ERAS implementation can have a transformative effect on team morale and productivity. Comprehensive training equips healthcare professionals with the necessary skills and knowledge to effectively implement ERAS protocols, fostering a sense of empowerment and confidence in their roles (33). In addition, ongoing educational opportunities allow staff to stay abreast of the latest advances in perioperative care and continuously improve their practice.

Our results showed that all healthcare professionals working with ERAS are convinced that the program is highly beneficial for patients undergoing surgery. This recognition underscores the potential of ERAS to improve patient outcomes in a wide range of surgical populations (34). However, while the benefits of ERAS are clear, the successful implementation of such programs requires the collective effort and commitment of all healthcare professionals involved.

Involving a wide range of healthcare professionals in the development and implementation of ERAS guidelines is crucial for several reasons. First and foremost, it fosters a sense of ownership and inclusiveness among the healthcare team. When nurses, physiotherapists, and other allied health professionals are actively involved in the decision-making process, they are more likely to feel invested in the success of the ERAS program. This sense of ownership can lead to increased motivation, collaboration, and overall success in implementing ERAS protocols (8).

Furthermore, including a variety of healthcare professionals in ERAS implementation promotes a holistic approach to patient care. Each member of the healthcare team brings unique perspectives, skills, and expertise to the table. By collaborating across disciplines, healthcare professionals can identify and address patient needs more comprehensively, leading to improved outcomes and patient satisfaction (35).

Moreover, providing background information and opportunities for professional development empowers healthcare professionals to contribute effectively to ERAS initiatives. Education and training sessions not only enhance clinical knowledge but also foster a deeper understanding of the rationale behind ERAS protocols. This understanding encourages healthcare professionals to actively engage in the implementation process and adapt their practices to align with ERAS principles (32).

By addressing these issues, healthcare organizations can overcome challenges and optimize the implementation of ERAS protocols, ultimately improving patient outcomes and enhancing the quality of perioperative care.

The feedback from this study has been instrumental in driving changes within our hospital's ERAS implementation. Specifically, several of the recommendations made by study participants, such as enhancing communication strategies and refining interprofessional collaboration, have led to changes in the clinical environment. For example, regular ERAS meetings for the entire ERAS team have been established to ensure consistent communication and alignment across disciplines, providing a platform for healthcare professionals to discuss ERAS-related matters and share updates. Additionally, the role of the ERAS nurse has been further emphasized, with the inclusion of a second ERAS nurse to improve coordination and ensure more comprehensive support throughout the perioperative care pathway. Clear Standard Operating Procedures (SOPs) have been further developed and standardized, ensuring consistency and clarity in the implementation of ERAS protocols. These changes reflect a concerted effort to optimize team collaboration, improve communication, and streamline the ERAS process, ultimately enhancing patient outcomes and aligning with the insights gathered from this study.

### 4.1 Limitations

Although this study provides valuable insights into the perceptions and experiences of healthcare professionals regarding the implementation of ERAS protocols for minimally invasive heart valve surgery, several limitations should be acknowledged.

First, the qualitative nature of the research methodology limits the generalizability of the findings. Qualitative research focuses on in-depth exploration rather than broad representation, and the perspectives captured in this study may not fully reflect the diversity of healthcare professionals or patient populations in other settings.

Second, the sample composition of the sample may have introduced bias into the study. Study participants were drawn from a single university hospital, and the inclusion of healthcare professionals with varying levels of experience and involvement in ERAS implementation may have influenced the interpretation of results. Additionally, the uneven representation of genders and professional roles within the sample warrants consideration, as this may have skewed the perspectives captured in the study.

Third, the retrospective nature of the study design may have introduced recall bias, as participants were asked to reflect on their experiences with ERAS implementation. Moreover, the experiences of healthcare professionals who joined the institution after the implementation of ERAS protocols may differ from those who were involved in the process from the beginning, potentially affecting the richness and diversity of perspectives.

### 4.2 Future research

To further advance the understanding of ERAS implementation beyond cardiac surgery, it is imperative to recognize the crucial role of health care providers and include their perspectives in future research efforts. Standardized assessments could capture specific challenges, such as staffing constraints and time pressure, to quantify their prevalence and impact. For instance, using quantitative surveys alongside qualitative methods would enable a more robust understanding of the barriers faced by healthcare workers.

Longitudinal studies are needed to evaluate the long-term impact of implementing ERAS protocols on patient outcomes and healthcare resource utilization. Plan-do-study-act cycles could be used to iteratively refine and optimize protocol implementation based on ongoing feedback and evaluation, allowing for continuous improvement and adaptation to changing healthcare contexts. Collaboration across institutions can capture a comprehensive understanding of ERAS implementation practices and challenges. Furthermore, evaluation of the financial implications, workload distribution, and organizational readiness for sustained ERAS programs is essential.

These evaluations should involve input from various health professionals, including nurses, physiotherapists, psychosomatic counsellors, and other allied health professionals, to ensure a comprehensive understanding of the challenges and successes of ERAS implementation.

Further exploration of alternative implementation strategies and interventions is needed to address existing barriers. Investigating technology-enabled solutions, innovative care delivery models, and interprofessional teamwork approaches will enhance effectiveness of ERAS. Engaging healthcare professionals from multiple disciplines in strategy development and evaluation will ensure inclusive and holistic implementation efforts.

## 5 Conclusion

In conclusion, effective communication strategies, interprofessional collaboration, clear guidelines, and the involvement of ERAS experts are essential to the successful implementation and maintenance of ERAS protocols. By addressing these issues, healthcare organizations can overcome challenges and optimize ERAS implementation, ultimately improving patient outcomes and enhancing the quality of perioperative care. Future research should focus on longitudinal studies to evaluate the long-term impact of ERAS implementation on patient outcomes and healthcare resource utilization, collaboration across institutions to gain a comprehensive understanding of ERAS implementation practices and challenges, and further exploration of implementation strategies and interventions to address existing barriers. Engaging healthcare professionals from multiple disciplines will ensure inclusive and holistic implementation efforts, ultimately advancing the field of ERAS and improving patient care.

## Data Availability

The raw anonymized data supporting the conclusions of this article will be made available by the authors, upon reasonable request.
